# Long-Term Results of Partial Breast Irradiation After Breast-Conserving Surgery for Early Stage Breast Cancer: A Prospective Phase II Trial in China

**DOI:** 10.3389/fonc.2020.550950

**Published:** 2020-09-02

**Authors:** Yan Li, Lin Shui, Xiaodong Wang, Yu Sun, Renming Zhong, Pixian Shui, Nianyong Chen

**Affiliations:** ^1^Radiation Oncology, Lung Cancer Center and State Key Laboratory of Biotherapy West China Hospital, Sichuan University, Chengdu, China; ^2^Department of Medical Oncology, Cancer Center, West China Hospital, Sichuan University, Chengdu, China; ^3^Department of Breast Surgery, West China Hospital, Sichuan University, Chengdu, China; ^4^Radiation Oncology, Cancer Center and State Key Laboratory of Biotherapy, West China Hospital, Sichuan University, Chengdu, China; ^5^School of Pharmacy, Southwest Medical University, Luzhou, China

**Keywords:** partial breast radiotherapy, 10-year follow-up, local control, cosmetic effects, late side events, breast cancer

## Abstract

**Background:**

Partial breast radiotherapy (PBI) has emerged as an option after breast-conserving surgery for early stage breast cancer patients.

**Methods:**

A total of 55 patients with early stage breast cancer between 2009 and 2013 were enrolled in this single-institutional phase II prospective clinical trial. All patients received adjuvant PBI-IMRT after lumpectomy, with the prescription of 48 Gy in 12 fractions at 4 Gy per fraction, 5 days a week. The primary endpoint was ipsilateral breast tumor recurrence (IBTR), the other endpoints were a regional nodal failure (RNF), distant metastasis (DM) rate, disease-free survival (DFS), and overall survival (OS). These endpoints were used to evaluate clinical outcomes. The cosmetic effects and the late toxicity were assessed according to Harvard standard scale and CTCAE 3.0, respectively.

**Results:**

In our cohorts, the median age was 45.60 years old (range 31–65 years) and 29.09% of these patients were post-menopause (*n* = 16). Most patients were T1 stage (65.45%) or N0 stage (70.91%). 80% of patients were ER-positive, 67.27% PR positive, and 61.82% HER2 negative. At the median follow-up of 9.25 years, RNF was 0% and IBTR occurred in only one patient (1.82%) to the chest wall. Except for one patient (1.82%) had DM to lung and pleura and died from disease progression, the remaining patients were alive at the end of the 10-year follow-up. The 10-year DFS and OS were 94.55 and 98.18%. One patient (1.82%) was diagnosed with endometrial cancer after PBI. Except for 9 patients who declined the cosmetic assessment, the rest of the 46 patients (83.64%) were all rated as good and well-satisfied with the appearance of the irradiated breast. No breast retraction and fibrosis were observed in any of the patients. Additionally, only 4 patients experienced grade 1 late toxicity (7.28%). None had grade 3 or higher late toxicity.

**Conclusion:**

This is the first study to report the 10-year results of PBI after breast-conserving surgery in Chinese patients. Our study suggested that PBI had durable local control and maintained good cosmetic outcomes with minimal late toxicity at long term follow up for the early stage breast cancer patients.

## Introduction

Breast cancer is the most common cancer in females worldwide (1). In China, it accounts for 17% of all malignancies in females (2). With more attention to breast health and the prevalence of mammogram screening, more and more patients were diagnosed at an early stage. The 5-year survival of early stage breast cancer after treatments is more than 90% (3). Over the past decades, the therapeutic strategy of limited-stage breast cancer has evolved. For example, breast-conserving surgery replaces the traditional radical mastectomy for the majority of the cases (4).

Conventional adjuvant radiation therapy after breast-conserving surgery is whole breast irradiation therapy (WBI) to avoid the recurrence of the remnant intact breast. WBI was typically delivered with standard fractionation over 5-week radiation of the entire breast with or without an additional 1 or 2 weeks of lumpectomy boost to the tumor bed. WBI involves the entire breast, adjacent skin, and even part of the heart and lung, which may increase the adverse events and influence the quality of life (5). Additionally, a large number of studies have shown that 60–80% of local recurrence after breast-conserving surgery was located within 3 cm from the primary tumor bed (6). Compared with WBI, partial breast radiotherapy (PBI) or accelerated partial breast radiotherapy (APBI) delivered a higher dose to the tumor bed with a margin in a shorter treatment course without affecting the surrounding normal tissues. This potentially improved the cosmetic outcomes and limit the short term and long term side effects. With shorter therapy course, APBI may also facilitate subsequent adjuvant therapy and reduce the medical costs. In carefully selected patients with early stage breast cancer, PBI, or APBI were shown to be not inferior to WBI (7) and emerged as alternative options following breast-conserving surgery. A study conducted by Coles showed that, at a median follow-up of 5 years, PBI was non-inferior to WBI with fewer late adverse events in the PBI group (8). However, there is limited data about the long-term results of PBI in the existing literature.

In addition, the characteristics of breast cancer in China are different from those in foreign countries, such as the younger median age and smaller breast volume in Chinese patients. Given a large number of patients and insufficient irradiation apparatus, once-a-day irradiation is more feasible than twice a day, which is commonly utilized in APBI. Whelan et al. compared the external beam APBI and WBI and found that APBI was non-inferior in preventing IBTR but associated with increased late toxicity and adverse cosmesis, which may be attributed to the twice-daily treatment (9). The results of this study provided the feasibility of PBI. However, PBI in China is still investigational and not recommended outside of clinical trials. There is evidence in radiobiology supporting a higher dose per fraction in PBI may enhance the cure rate without more late toxicities. However, the appropriate irradiation technique, dose per fraction, the treatment modality, and the selection of a potential population of PBI need to be further evaluated in clinical trials. As preliminary evidence for the development of PBI, we conducted a prospective phase II study and provided our 10-year follow-up outcomes and late toxicities of PBI in Chinese patients with early stage breast cancer.

## Materials and Methods

### Patient Population

The study was designed as a single-arm, single-center, phase II clinical trial to evaluate the 10-year results of the safety and efficacy of PBI after breast-conserving surgery. Due to the rate of breast conservation in China was very low at that time, only small sample clinical studies were conducted. A total of 55 patients with early stage breast cancer received lumpectomy and PBI in West China Hospital of Sichuan University were enrolled from 2009 and 2013. The study protocol was approved by the hospital institutional review board and ethics committee. All the study participants signed informed consent.

The eligibility criteria for enrollment in this clinical trial were patients at least 30 years old, histologically confirmed invasive breast cancer or ductal carcinoma *in situ* (DCIS), underwent axillary lymph node dissection or sentinel node biopsy, a lesion no larger than 3 cm with a negative surgical margin greater than 2 mm (in case of pure DCIS ≥ 5 mm) were required; no distant metastasis or contralateral breast cancer. Patients were excluded from the protocol if they had extensive intraductal carcinoma, involved/unknown margins (R1/Rx), or pregnant.

### Treatment Planning

Details of radiation technique and treatment have been published elsewhere (10). Due to the smaller average breast volume of Chinese patients, we defined clinical target volume (CTV1) as the excision cavity volume, architectural distortion, lumpectomy scar, seroma, and surgical clips expanding 1 cm circumferentially out uniformly. CTV2 was defined as the area expanding 1 cm uniformly out from CTV1. Some studies had reported poor cosmetic outcomes in the group of APBI with 3.85 Gy per fraction, twice a day for 1 week consecutively for a total of ten treatments with three-dimensional conformal radiotherapy (11). Some possible reasons for poor cosmesis may be dose heterogeneity of the three-dimensional conformal plan, or the normal breast irradiation volume is too large. Another possible explanation could be that a twice-daily schedule used in these studies may have a greater biological effect due to incomplete normal tissue repair (12).

Intensity-modulated radiotherapy (IMRT) had the advantage of improving the dose conformality compared to the 3D technique and with more normal tissue sparing. Therefore, in our studies, all the patients received IMRT. The median age of breast cancer patients in China is younger, but the tumor bed often requires a higher dose of radiation in younger patients. Based on the protocol from tumor bed boost irradiation, the total dose to CTV1 was 48 Gy in 12 fractions over 16 days (13). The total dose to CTV2 was 41.6 Gy in 12 fractions over 16 days (i.e., the dose per fraction is 3.47 Gy). Using the linear-quadratic model and assuming an alpha/beta ratio of three, the prescription of CTV1 was equivalent to 66 Gy, and the prescription of CTV2 was equivalent to 54 Gy in standard 2-Gy fractionation. PTV was generated to account for set-up uncertainties and organ motion by 10 mm automatic expansion of the CTV (14). The treatment planning for APBI was evaluated according to RTOG-0319 (15). We made slight adjustments individually based on different techniques and prescriptions. The following constraints were utilized for plan optimization: PTV coverage, 100% of PTV covered by 95% of the prescribed dose (45.6 Gy, 39.5 Gy); maximal dose to PTV < 105% (50.4 Gy,43.7 Gy); minimal dose to PTV < 93% (44.6 Gy,38.7 Gy);uninvolved breast, not >60% received 20 Gy, not >40% received 40 Gy (V20 < 60%, V40 < 40%); ipsilateral lung, not >20% received a dose >10 Gy (V10 < 20%); contralateral lung, not >10% received a dose >5 Gy (V5 < 10%); contralateral breast, not >5% received 5 Gy (V5 < 5%), heart, not >10% received 5 Gy (Table 1).

**TABLE 1:** Dose–volume constraints/normal tissue tolerances.

PBI	Dosimetric parameter	Dose–volume constraints
PTV1	48 Gy	Dmax < 50.4 Gy, Dmin > 44.6 Gy
PTV2	41.6 Gy	Dmax < 43.7 Gy, Dmin > 38.7 Gy
Breast-PTV	V20	<60%
(uninvolved breast)	V40	<40%
Contralateral breast	V5	<5%
Ipsilateral lung	V10	<20%
Contralateral lung	V5	<10%
Heart	V10	<5 Gy

### Follow-Up, Endpoints and Cosmetic Evaluation

Follow-up ended in October 2019. Patients were evaluated every 6 months in the first 5-year for routine follow-up and then once yearly with physical examination and imaging. Suspicious abnormalities were required for pathological confirmation. The primary objective of this study is to prospectively evaluate the long-term efficacy and late side-effects of PBI after breast-conserving surgery in early stage breast cancer. The primary endpoint was local control, which was evaluated by ipsilateral breast tumor recurrence (IBTR). Other clinical outcomes that were evaluated were a regional nodal failure (RNF), distant metastasis (DM), disease-free survival (DFS), and overall survival (OS).

Cosmetic effects were evaluated qualitatively (subjectively) and quantitatively (objectively) at each follow-up. The qualitative cosmetic results, including the appearance of the surgical scar, size, fibrosis, and shape of the treated breast, as well as skin color, location and shape of the areola and nipple, were rated as excellent, good, fair, and poor by the physician (16) using the Harvard scale. “Excellent” means there is no difference between the treated breast and the contralateral breast, and “good” was regarded as a little difference existing between them, “fair” as significant difference without severe deformation, “poor” as severe deformation. For quantitative evaluation, breast retraction was used to assess the extent of the breast deformation, which was measured by the lateral and vertical displacement of the nipple compared to the contralateral breast (17).

Late effects were evaluated according to the Common Terminology Criteria for Adverse Events (CTCAE) version 3.0 at the time of follow-up by the treating physician.

### Statistical Analysis

Microsoft Excel was used to record and calculate the value, median, ranges, or percentage for patient characteristics. The estimated likelihoods for IBTR, RNF, DM, DFS, and OS were calculated using the Kaplan-Meier method using STATA version 13.

## Results

The clinical and demographic characteristics of enrolled patients were summarized in Table 2. The median age was 45.60 years old (range 31–65 years) and 29.09% of these patients were post-menopause (*n* = 16). Most patients were T1 stage (65.45%) or N0 stage (70.91%). Regarding the histology, 90.91% of patients were infiltrating ductal carcinoma, 80% ER-positive, 67.27% PR-positive, 61.82% HER2-negative, and 12.73% triple-negative breast cancer. All patients had a negative surgical margin. One patient (1.82%) had vascular cancer embolus. 47 patients (85.45%) received adjuvant chemotherapy, 81.82% of all patients received hormonal therapy and only 2 patients (3.64%) received anti-HER2 therapy.

**TABLE 2:** Clinical and demographic baseline characteristics of patients in this study.

Clinical and demographic baseline characteristics of patients (*n* = 55)		
**Age**		**year**

Median		45.60
Range		31–65

**Menopausal status**	***n***	**(%)**

Postmenopause	16	29.09
Premenopause	39	70.91

**T stage**	***n***	**(%)**

T0	1	1.82
T1	36	65.45
T2	18	32.73

**N stage**	***n***	**(%)**

N0	39	70.91
N1	13	23.64
N2	3	5.45

**Histologic type**	***n***	**(%)**

Infiltrating ductal carcinoma	50	90.91
Infiltrating lobular carcinoma	1	1.82
Mucinous carcinoma	4	7.27

**Hormone receptor status**	***n***	**(%)**

ER positive	44	80.00
ER negative	11	20.00
PR positive	37	67.27
PR negative	15	27.27
PR unknown	3	5.45
Her2/neu positive	20	36.36
Her2/neu negative	34	61.82
Her2/neu unknown	1	1.82
ER-/PR-/Her2/neu-	7	12.73

**Ki-67**	***n***	**(%)**

<15%	19	34.55
15–50%	24	43.64
>50%	4	7.27
Unknown	8	14.55

**Vascular cancer embolus**	***n***	**(%)**

Negative	54	98.18
Positive	1	1.82
**Adjuvant therapies**		

**Chemotherapy**	***n***	**(%)**

Received	47	85.45
Not received	8	14.55

**Hormone therapy**	***n***	**(%)**

Received	45	81.82
Not received	10	18.18

**Target therapy**	***n***	**(%)**

Received	2	3.64
Not received	53	96.36

*ER, estrogen receptor; PR, progesterone receptor; HER2, human epidermal growth factor receptor 2.*

The median time of follow-up for all patients was 9.25 years (range 6.92–10.67 years). The failure patterns during the 10-year follow-up were shown in Table 3. For the 55 patients, there was no RNF (0%). However, one patient (1.82%) had IBRT 1 year after the completion of PBI. One patient (1.82%) experienced a second primary endometrial cancer after PBI. One patient (1.82%) had DM to lung and pleura nearly 3 years after PBI therapy and died from tumor progression 2 years later. The remaining patients were alive at the end of the follow-up. The 10-year DFS and OS were 94.55 and 98.18%, respectively. The two patients who presented with local recurrence or DM were relatively high-risk, such as triple-negative breast cancer or high Ki-67.

**TABLE 3:** The clinical outcomes of the patient cohorts in our study.

Clinical outcomes (*n* = 55)	
Failure pattern	10-year control rate (*n* [%])
Ipsilateral breast tumor recurrence	1 (1.82%)
Regional nodal failure	0
Distant metastasis	1 (1.82%)
Disease-free survival	52 (94.55%)
Overall survival	54 (98.18%)

The overall cosmetic results from radiation were assessed by the same radiation oncologist at approximately 6 months of follow-up. Nine patients who did not respond to this evaluation. At a median follow up of 9.25 years, 46 patients (83.64%) were evaluated as good by the radiotherapist. And the 46 patients were satisfied with their overall cosmetic effects as good (83.64%). In other words, 83.64% of patients did not have breast retraction post radiation, both subjectively and objectively (Table 4).

**TABLE 4:** The overall cosmetic effect evaluation of our patient cohorts.

	*n*	(%)
**Cosmetic effect evaluation (*n* = 55)**		
Excellent	0	0
Good	46	83.64%
Fair	0	0
Poor	0	0
No response	9	16.36%
**Satisfaction with treatment (*n* = 55)**		
Excellent	0	0
Good	46	83.64%
Fair	0	0
Poor	0	0
No response	9	16.36%
**Breast retraction (*n* = 55)**		
Yes	0	0
No	46	83.64%
No response	9	16.36%

Four patients enrolled in this study experienced side effects during the 10-year follow-up (7.28%), and the toxicities observed all were grade 1 events (Table 5). No grade 3 or higher adverse events were recorded. In detail, hyperpigmentation, mild atrophy of the treated breast, alopecia, and impaired lung function were observed in one patient (1.82%), respectively.

**TABLE 5:** The 10-year late side-events occurred in our patient cohorts.

10-year prevalence of late side-effects (*n* = 55)				
Toxicity (*n*)	Grade 1	Grade 2	Grade 3	Grade 4
Hyperpigmentation	1	0	0	0
Atrophy	1	0	0	0
Alopecia	1	0	0	0
Lung function	1	0	0	0

## Discussion

To our knowledge, this is the first phase II study reporting the 10-year results of clinical efficacy and the late effects of PBI after breast-conserving surgery in Chinese patients. Currently, limited data demonstrated long-term outcomes of PBI. Table 6 listed the outcomes of current studies with a 10-year follow-up of APBI after breast-conserving therapy. Through comparison with other studies, we found that the results of our study are in line with the results presented by other cooperative groups. Although one patient experienced recurrence to the chest wall, she was identified as a high-risk patient due to a high value of Ki-67% (40–45%) and HER2 overexpression but without anti-HER2 treatment. The rate of DM was 1.82% and the OS of the cohorts in our study was 98.18%. One patient died of metastases to the lung and pleura, who was triple-negative with a high risk of recurrence. From the results of our study, it was suggested that for the early stage breast cancer patients, PBI had durable local control and maintained good cosmesis at long-term follow up.

**TABLE 6:** The comparison of clinical outcomes, cosmetic effects and late side events at 10-year follow-up between our study and other studies.

Comparison of efficacy, cosmetic effects and side events (at 10 years)						
Study	Phase	Treatment	Patients	Therapeutic effect	Cosmetic effect evaluation	Side effects
Becherini et al. (33)	III	30 Gy in 5 non-consecutive fractions	22 DCIS	IBTR/DM 0; OS 90.9%	Not mentioned	Late toxicity:0
White et al. (34)	I/II	34 Gy in 10 twice-daily fractions over 5 days for HDR; 45 Gy in 3.5–5 days for LDR brachytherapy	100 patients, unifocal, <3 cm, surgical margins (-), 0–3 positive axillary nodes without extracapsular extension	IBTR 5.2%, RR 3.1%, DM 0; DFS 69.8%, OS 78.0%	Not mentioned	Not mentioned
Polgar et al. (35)	III	7 × 5.2 Gy HDR multi-catheter brachytherapy (*n* = 88) or 50 Gy electron beam irradiation (*n* = 40)	128 patients, pT1 pN0-1 M0, grade 1–2, non-lobular breast cancer, negative surgical margins	LR 5.9%; OS 80%, CSS 94%, DFS 85%	81% was rated as excellent or good	Not mentioned
Vicini et al. (28)	III	34 Gy with brachytherapy or 38.5 Gy with external beam, 3DCRT in 10 fractions, twice daily at least 6 h apart	2,107 heterogeneous early stage patients	IBTR 4.6%, OS 98%	Not mentioned	Grade 1: 845 (40%), grade 3: 201 (10%)
Wobb et al. (36)	Matched-pair analysis	Interstitial or balloon brachytherapy	481 early patients	IBTR 4%, RR 1%, contralateral breast failure 3%, DM 6%;DFS 91%, OS 75%	95% was rated as excellent or good	Not mentioned
Kamrava et al. (37)	Retrospective study	Interstitial multicatheter brachytherapy.	1,356	IBTR 7.6%, RNF 2.3%, DM 3.8%, CCS 96.3%, OS 86.5%, new contralateral cancers 4.6%	Excellent or good: 84% of patients with >5 years of follow-up.	Not mentioned
Our study	II	IMRT PBI, 48 Gy/12 fractions, one-a-day, 4 Gy per fraction, 5 consecutive weekdays	55 heterogeneous early stage patients	IBTR 1.82%, DM 1.82%, RNF 0, DFS 94.55%, OS 98.18%	Excellent or good: 83.64%	Grade 1: 4 (7.28%), grade 3 or higher: 0

*IBTR, ipsilateral breast-tumor recurrence; DM, distant metastasis; RNF, regional node failure; LR, local recurrence; RR, regional recurrence; DFS, disease-free survival; CCS, cause-specific survival; OS, overall survival; HDR, high-dose-rate; LDR, low-dose-rate.*

In western countries, a large number of studies have compared the difference between WBI and APBI after breast-conserving surgery. RTOG 0319 was a phase I/II study that evaluated the efficacy and safety of APBI 3D-CRT, in which the 4-year IBTR, DFS, and OS were 6, 84, and 96%, respectively. Moreover, RTOG 0319 showed that 4% of patients had grade 3 adverse reactions (18). Livi et al. (19) randomly assigned 520 patients to receive WBI or APBI-IMRT treatment after breast-conserving surgery. The radiation doses of the two groups were 50 Gy in 25 fractions plus 10 Gy in 5 fractions of boost therapy for WBI and 30 Gy in 5 fractions for APBI, respectively. The results of 5-year follow-up showed that there was no significant difference between the two groups in IBTR and OS, but APBI-IMRT had significantly better performance in acute and chronic toxicities and cosmetic effects. Bitter et al. (20) enrolled 242 patients with breast cancer and found that the chronic lymphedema, self-confidence, and fatigue in the APBI group were significantly less than those in the WBI group in the 1-year follow-up. In the second year, the cosmetic effects of breast appearance in the APBI group were significantly better than those in the WBI group, and there was no significant difference in the recurrence rate between the two groups.

A meta-analysis (21) comparing APBI and WBI in 1,140 patients showed that there was no significant difference in DM, lymph node recurrence, and OS between patients receiving APBI and WBI. According to traditional perspectives, hypofractionated radiotherapy may lead to tissue fibrosis, telangiectasia, and pain. Some studies have shown that radiation spillage to lung decreased significantly at the middle to the high dose of APBI-IMRT, even if IMRT may lead to more exposure to lung at the dose of 2.5–5 Gy, the cumulative dose of heart was significantly reduced, which may benefit patients with left-sided breast cancer (22, 23). TARGIT study (24, 25) found the cardiac mortality of patients with partial breast irradiation was significantly lower than that of the whole breast irradiation group. Furthermore, recent studies have shown that there was no positive correlation between the target dose and long-term adverse effects. At the median follow-up of 4.8 years, 80% of the patients treated with APBI were rated as excellent or good cosmetic outcomes (26). In addition, APBI was suggested to reduce medical costs. A study conducted by Shah et al. (27) has shown that APBI 3D-CRT may save 6 million dollars compared with WBI for the treatment of 1,000 patients, versus 2 million dollars for APBI-IMRT. However, these studies were controversial. A randomized controlled study comparing the safety and cosmetic effects of 3D-CRT and WBI found that APBI 3D-CRT presented increased cosmetic and late radiation toxicity compared with standard WBI at 3-year follow-up (11). RTOG 0413, a phase III trial comparing different APBI technique with WBI for a diverse group of early stage patients, have shown that APBI did not meet the equivalent criteria of WBI at a median follow-up of 10.2 years, even though the absolute difference of IBTR between the two groups was only 0.7% difference (28). The 5-year recurrence rate of partial breast irradiation in the ELIOT study was slightly higher than that of the whole breast irradiation group (29). Further analysis found that high-risk factors of partial breast irradiation for tumor recurrence were the lesion greater than 2 cm, axillary lymph node metastasis number greater than 4, histological grade 3, triple-negative type, the 5-year recurrence rate of patients between at least one or more risk factors and no risk factors were 11.3 and 1.5% (*p* < 0.0001), which suggested the patients with the above risk factors may not be suitable for the partial breast irradiation. RAPID research found the cosmetic effects of partial breast irradiation group were inferior to the whole-breast irradiation group (11). In addition, a retrospective study found the ipsilateral axillary recurrence rate of partial breast irradiation increased than that of whole-breast irradiation after long term follow-up (30).

Although non-inferiority and better cosmetic effects of APBI were seen mostly in current clinical trials composed of low-risk patients, long-term follow-up and larger-scale clinical trials are needed to determine whether WBI can be completely replaced by PBI after breast-conserving surgery in early stage breast cancer.

Patient selection for APBI remains controversial. Given APBI focused on the local control rate, not all patients after breast-conserving surgery is a candidate for APBI. With the successful development of several randomized clinical trials, ASTRO updated the APBI guidelines in 2018 (31), which clarified the APBI criteria for patients: (1) age: patients ≥50 years old can be included in the “suitable” group to receive APBI without participating in clinical trials. Patients between 40 and 49 years old are classified as the “cautionary” group, and patients <40 are categorized as “unsuitable” group; (2) negative surgical margin: ≥2 mm; (3) Tis and tumor diameter less than 3 cm in stage T1 and T2 are grouped in “cautionary”; (5) ER-positive; (6) N0 stage confirmed by sentinel lymph node biopsy or axillary lymph node dissection; (7) single lesion. Due to the differences in eligibility criteria and the development of radiation technologies, the results of APBI between different countries may be different. Therefore, research needs to take into account the characteristics of breast cancer in China. Studies need to select the appropriate population with the optimal radiotherapy equipment and modality to obtain meaningful data in China to guide clinical practice. So far there are no similar criteria for Chinese patients. Most studies conducted in China use the same criteria for enrollment as international research, mainly including low-risk patients. However, relatively heterogeneous patients were enrolled in our study, including patients from “suitable,” “cautionary” and “unsuitable” groups from the ASTRO updated consensus guidelines. Our results showed that the 10-year IBTR, DM, and RNF were still very low in these patients even some patients are relatively higher risk. Our study may provide evidence for the establishment of unique criteria for potential patients of PBI in China.

Aside from the excellent local control, we also have shown the 10-year results of cosmetic effects and late side-events of PBI. Except for 9 patients who did not respond to the cosmetic evaluation, the rest of the 46 patients (83.64%) were rated as good according to Harvard standard, and they were well-satisfied with the cosmesis of the treated breast. None of them had breast retraction. There were only 4 patients in our study have experienced grade 1 late side effects (7.28%) and no patients had grade 3 or higher side events. A randomized clinical trial of phase III also confirmed the feasibility of APBI-IMRT. The grade 1 and 2 acute toxicity of APBI group and WBI group were 5% vs 22% and 0.8% vs 19%, respectively (32). Given fewer acute toxicity of APBI, there was limited data regarding 10-year follow-up monitor tumor recurrence rates and incidence of late effects.

The limitation of our study design was that it was not randomized against WBI, which remains the standard of care. Therefore it is not possible to draw a conclusion on the effectiveness, safety, and feasibility of PBI to WBI. In addition, this is a single institution study with a small sample size which is another limitation of this trial.

In conclusion, there is still unsettlement with PBI that needs to be further investigated. For example, the current technique and dose of PBI remain controversial. The other debate is the definition of the target volume in PBI. We believe that with the development of radiation technique and the maturation of the existing clinical trials with longer follow up, the PBI has the potential to provide a novel option for breast-conserving patients with early stage breast cancer. This is the first study to explore the long-term results and the safety and efficacy of PBI in Chinese patients. As preliminary evidence of the ongoing phase III clinical trial, our study may provide potential guidelines to the application of PBI in Chinese patients.

## Data Availability Statement

The raw data supporting the conclusions of this article will be made available by the authors, without undue reservation.

## Ethics Statement

The study protocol was approved by the Institutional Review Board of West China Medical Center and all the study participants signed the informed consent.

## Author Contributions

YL and LS contributed equally to this work. All authors contributed to the article and approved the submitted version.

## Conflict of Interest

The authors declare that the research was conducted in the absence of any commercial or financial relationships that could be construed as a potential conflict of interest.
